# Developmental dynamics of neurotensin binding sites in the human hypothalamus during the first postnatal year

**DOI:** 10.3389/fncel.2014.00251

**Published:** 2014-09-10

**Authors:** Mohamed Najimi, Alain Sarrieau, Nicolas Kopp, Fatiha Chigr

**Affiliations:** ^1^Biological Engineering Laboratory, Life Sciences, Sultan Moulay Slimane UniversityBeni-Mellal, Morocco; ^2^Unité de Formation et de Recherche de Biologie, Université de Bordeaux 1, TalenceFrance; ^3^Lyon 1 UniversityLyon, France

**Keywords:** neurotensin receptor, human hypothalamus, newborn brain, infant brain development, autoradiography

## Abstract

The aim of the present study was to determine a detailed mapping of neurotensin (NT) in the human hypothalamus, during the first postnatal year using an *in vitro* quantitative autoradiography technique and the selective radioligand monoiodo-Tyr3-NT. Ten human postmortem hypothalami obtained from control neonates and infants (aged from 2 h to 1 year of postnatal age) were used. The biochemical kinetics of the binding in all obtained in this study revealed that the binding affinity constants were of high affinity (in the nanomolar range) and did not differ significantly between all cases investigated. Furthermore, competition experiments show insensitivity to levocabastine and were in favor of the presence of the high affinity site of NT receptor. Autoradiographic distribution showed that NT binding sites were widely distributed throughout the rostrocaudal extent of the hypothalamus. However, the distribution of NT binding sites was not homogenous and regional variations exist. In general, the highest densities were mainly present in the anterior hypothalamic level, particularly in the preoptic area. High NT binding site densities are also present at the mediobasal hypothalamic level, particularly in the paraventricular, parafornical, and dorsomedial nuclei. At the posterior level, low to very low densities could be observed in all the mammillary complex subdivisions, as well as the posterior hypothalamic area. Although this topographical distribution is almost identical during the postnatal period analyzed, age-related variations exist in discrete structures of the hypothalamus. The densities were higher in neonates/less aged infants than older infants in preoptic area (medial and lateral parts). The developmental profile is characterized by a progressive decrease from the neonate period to 1 year of postnatal age with a tendency to reach adult levels. On the other hand, the low levels of NT binding sites observed in posterior hypothalamus did not vary during the first postnatal year. They contrast in that with the very high levels we reported previously in adult. In conclusion, the present study demonstrates the occurrence of high NT binding sites density in various structures in many regions in the human neonate/infant hypothalamus, involved in the control of neuroendocrine and/or neurovegetative functions.

## INTRODUCTION

The brain-gut peptide neurotensin (NT) is a neurotransmitter and neuromodulator in the central nervous system (CNS), and acts as a hormone in the gastrointestinal tract (Hermans and Maloteaux, 1998). Previous studies have shown that NT has a role, in both central and peripheral nervous systems. NT-producing neurons and their projections are widely distributed in the CNS, which explains the wide range of effects of this peptide (for review see Rostène and Alexander, 1997). The neuropeptide acts via three recognized receptors: NT1, NT 2, and NT3 (Vita et al., 1993; Mazella et al., 1996; Martin et al., 2003; Dicou et al., 2004). NT1 and NT2, are seven transmembrane domain G protein-coupled receptors, whereas NT3 has a single transmembrane domain sorting receptor that is predominantly associated with vesicular organelles and shares 100% homology with gp95/sortilin (Vincent et al., 1999). From a neuroendocrine/ endocrine perspective, the neuropeptide and its receptors are mainly located in neuronal synaptic vesicles of hypothalamus (Rostène and Alexander, 1997), in adenohypophysis cells (Reyes et al., 2008) and in neuroendocrine cells of the small bowel where they are involved in enteric digestive processes, gut motility and intestinal inflammatory mechanisms (for review see Evers, 2006). Previous studies have shown the role of the peptide in the regulation of the hypothalamo–pituitary–adrenal gland axis (Geisler et al., 2006). The hypothalamic region is of particular interest in the study of neuroendocrine regulation and related endocrine and autonomic disorders due to its role in the control of hormonal release, funneling converging inputs from widely distributed vegetative/autonomic regions. Thus, intracerebroventricular or local NT injections in hypothalamus have been associated with dramatic alterations of the plasma levels of most anterior pituitary hormones (Alexander and Leeman, 1992; Vijayan et al., 1994; Rostène and Alexander, 1997; Sicard et al., 2005). Of interest, the hypothalamus, as reported in laboratory animals and human is especially enriched in endogenous peptide and NT receptors (Sarrieau et al., 1985; Kanba et al., 1986; Mai et al., 1987; Sakamoto et al., 1987; Rostène and Alexander, 1997; Najimi et al., 2014). The human hypothalamus is not completely mature at birth and its maturation continues during the postnatal period with relation to the maturation of peptidergic factors regulating pituitary function (Swaab, 1995). Transient nature of hormone levels seems to prevail for most of the first postnatal life. The stabilization of their rates and their hypothalamic regulatory systems is observed well after this period (Donovan, 1980) which makes postnatal period highly sensitive to structural or neurochemical alterations. These changes could perturb not only neuroendocrine circuits sub-serving the anterior pituitary, but indirectly disrupt widespread hypothalamic autonomic functions as well. Indeed, previous studies have shown that events on early life may have long-term effects on physiology (Contreras et al., 2013). Dysregulation of NT neuromodulation in many brain systems notably during development has been hypothesized to be involved in the pathogenesis of several insults. Changes in levels of NT and its cognate receptors have been reported in victims of sudden infant death syndrome (SIDS; Chigr et al., 1992; Coquerel et al., 1992). The relevance of the neurotensinergic system activity in the correct development and function of the human brain seems to be of great importance.

Developmental aspects of neurotensinergic system in human hypothalamic–pituitary complex are scare and concerned particularly the endogenous NT in developing human hypothalamus (Sakamoto et al., 1987) and pituitary (Reyes et al., 2008). Developmental principles in NT and NT receptors have been extensively reported in laboratory animals notably in murine brain (Palacios et al., 1988; Sato et al., 1991, 1992; Lépée-Lorgeoux et al., 2000). This relevant literature about the neuroanatomy of central NT and its receptors indicate the presence of different developmental profiles. Moreover, it has been suggested that NT could promote and sustain survival, and be involved in neuronal migration pattern and synapse formation (O’Leary and Koester, 1993). In addition, NT has been shown to promote dendrite elongation and dendritic spine maturation (Gandou et al., 2010). Finally, studies in non-neuronal cells have shown that NT exerts mitogenic and trophic effects in normal and cancer cells in liver, pancreas, lung, and prostate (Hasegawa et al., 1994; Sehgal et al., 1994). Although well studied in animals, the developmental characteristics of NT receptors have been poorly investigated in humans and no investigations have concerned the hypothalamus. As far as NT receptors are known to be important in the function of the neuropeptide, only few studies have specifically examined NT receptor development in human brain (the whole hemisphere: Zsürger et al., 1992 and medulla oblongata: Mailleux et al., 1990; Chigr et al., 1992). In this study, we investigated the developmental distribution of NT binding sites in normal post mortem human hypothalamus obtained from newborns and infants.

## MATERIALS AND METHODS

### SOURCE AND PREPARATION OF HUMAN TISSUES

We studied NT binding sites in the hypothalami of ten neonates and infants, autopsied at the Edouard Herriot and Lyon Sud Hospitals (Lyon, France) in accordance with written consent from the next of kin and local ethical approval (Ethics Committee of the two French laboratories). None of these subjects died as a result of neurologic, neuroendocrine, or endocrine disease (**Table 1**) and no pathological lesions were observed after macroscopic and microscopic examination of the brains. Ante mortem variables as age and sex were also summarized in **Table 1**. Furthermore, available information relating to tissue donor ante-mortem variables does not indicate the presence of agonal state or the presence of specific medication. At autopsy, the brains were removed from the cranium and the hypothalami were dissected out at 4°C by taking the parallel plane joining the optic chiasma and the anterior commissure as frontal plane and the caudal plane just behind the mammillary complex. The hypothalamus samples were immediately frozen at -80°C and stored at the same temperature until mounted on cryostat chucks. The frozen hypothalami were then sliced as 20 μm thick coronal sections at -20°C (Frigocut 2800, Reichert Jung, Heidelberg, Germany). Sections were collected onto chrome alum gelatin coated slides (Mentzel-Gläser, Braunschweig, Germany) and stored at -20°C until use. For the anatomical localization of the hypothalamic nuclei and areas, sections adjacent to those used for autoradiography were stained with cresyl violet. We adopted conventional nomenclature we used in our previous investigations (Najimi et al., 2001, 2006, 2014).

**Table 1:** Source of brain tissues.

Cases	Sex	Age	Post-mortem delay (h)	Cause of death
A	F	2 h	14	Pulmonary hypoplasia
B	M	35 h	34	Amniotic inhalation with gastric regurgitation
C	M	1 day	10	Oedematic alveolitis with Refractory hypoplasia
D	M	3 days	15	Diaphragmatic hernia
E	M	1 month	20	Liver lesions
F	M	1 month	5	Enterocolitis
G	F	2 months	24	Pulmonary hemorrhage
H	F	4 months	24	Kidney medullar invagination
I	M	6 months	22	Pneumopathy
J	F	1 year	7	Hepatic necrosis

### *IN VITRO* QUANTITATIVE AUTORADIOGRAPHY

The slides containing the sections of the hypothalamus were first warmed to room temperature and then incubated at 4°C for 2 h, with 0.1 nM monoiodo ^125^I-Tyr-NT (2000 Ci/mmol) in 50 mM Tris-HCl buffer (pH 7.5) containing 5 mM MgCl_2_, 0.2% bovine serum albumin and 0.02 mM bacitracin (New England Nuclear: NEN, Wellesley, MA, USA). Non-specific binding was determined as the binding of ^125^I-NT in the presence of 1 μM of unlabelled NT_1-13_. After incubation, the slides were washed with ice cold buffer four times for 2 min each and rapidly dried with cold air. Labeled sections and iodinated standards (Amersham, Courtaboeuf Cedex, France) were then apposed to 3H sensitive Ultrofilm (Amersham) in Amersham exposure cassettes. After 2 weeks exposure in dark conditions at 4°C, the film was developed in Kodak D19 (Eastman Kodak, Rochester, NY, USA) for 3 min, dipped in water and fixed with Kodak rapid fixer for 10 min.

Competition experiments were performed by incubating serial sections from the anterior and mediobasal hypothalamic levels in the same medium containing graded concentrations of unlabelled NT (10^-12^ to 10^-6^ M). IC_50_ values were calculated from inhibition curves as peptide concentrations inhibiting 50% of monoiodo ^125^I-Tyr-NT binding. Kinetics (IC_50_ and K_D_) analysis was computed by the method of Parker and Waud (1971).

Densitometric analysis of ^125^I-NT binding was carried out according to the methods described previously (Chigr et al., 1992) using a computer assisted image analysis system (Biocom 2000, les Ulis, France) and the standards coexposed. Values for total and non-specific binding of ^125^I-NT were obtained for each region by averaging four to eight readings for each hypothalamic nucleus and area on an individual autoradiograph.

#### Statistics

Results were expressed as means ± SEM. The differences of ^125^I–NT binding site densities between the different age periods analyzed were evaluated using the analysis of variance (ANOVA) with 99% significant level. When necessary, *post hoc* tests were performed and the data were analyzed using the Scheffe’ *F* test of the Stat View 512^++TM^ computer program.

## RESULTS

We firstly assessed the binding characteristics of the ^125^I-NT in the hypothalami of the cases examined. Computer analysis of binding isotherms showed that the apparent constant Kd and IC_50_ are in the range of 0.88-1.40 nM and 1.96-2.8 Nm, respectively (**Table 2**).

**Table 2:** *K*d values of [^**125**^I]-neurotensin _**1**-**13**_ binding and IC_**50**_ values (Inhibition of specific ^**125**^I–NT binding to hypothalamic sections by unlabeled NT) in different human individuals during the peri- and postnatal period.

Cases	Sex	Age	PMD	Kd (nM)	IC_50_ (nM)
A	F	2 h	14	1.40	1.9
B	M	35 h	34	0.91	2.6
C	M	1 day	10	0.88	2.7
D	M	3 days	15	1.45	2.3
E	M	1 month	20	0.98	2.8
F	M	1 month	5	1.10	2.6
G	F	2 months	24	1.20	2.6
H	F	4 months	24	0.96	1.9
I	M	6 months	22	1.30	2.4
J	F	1 year	7	1.10	2.4

*Note that neither sex nor age influence on the values obtained.*

### AUTORADIOGRAPHIC LOCALIZATION OF ^125^I-NT BINDING

**Figure 1:**
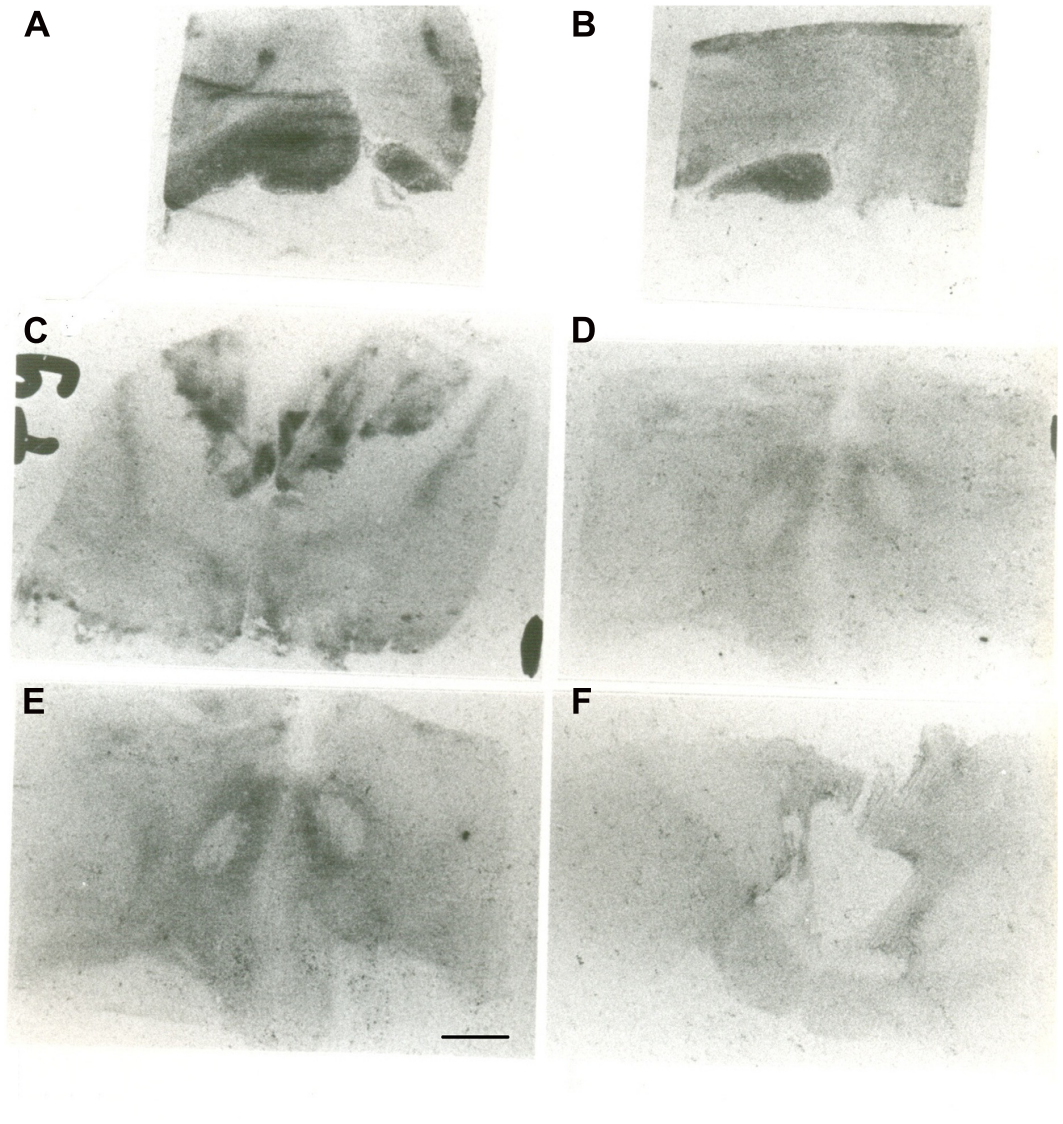
**Autoradiographic images of neurotensin (NT) binding sites in coronal hypothalamic sections obtained in human neonate.** This Figure represents the most rostral level of the anterior hypothalamus. **A** represents the edge of the rostral level at the diagonal band of Broca (septo-hypothalamic junction), and **B** represents the beginning of the hypothalamus *senso stricto* (preoptic area level). Note that at rostral level, the binding is higher in the preoptic region, whereas it decreases caudally at the anterior and dorsal areas **(C)**. **D,E** represent microphotographs obtained at the mediobasal level, illustrating the rostrocaudal variations of NT binding sites. **E** represents coronal section obtained more caudally, comprising the junction with the beginning of the posterior hypothalamus. **F** illustrates NT binding sites distribution in the posterior level of human neonate hypothalamus, illustrating the distribution of NT radiolabeling in the mammillary complex principally and surrounding structures. Scale bar = 3.5 mm.

**Figure 2:**
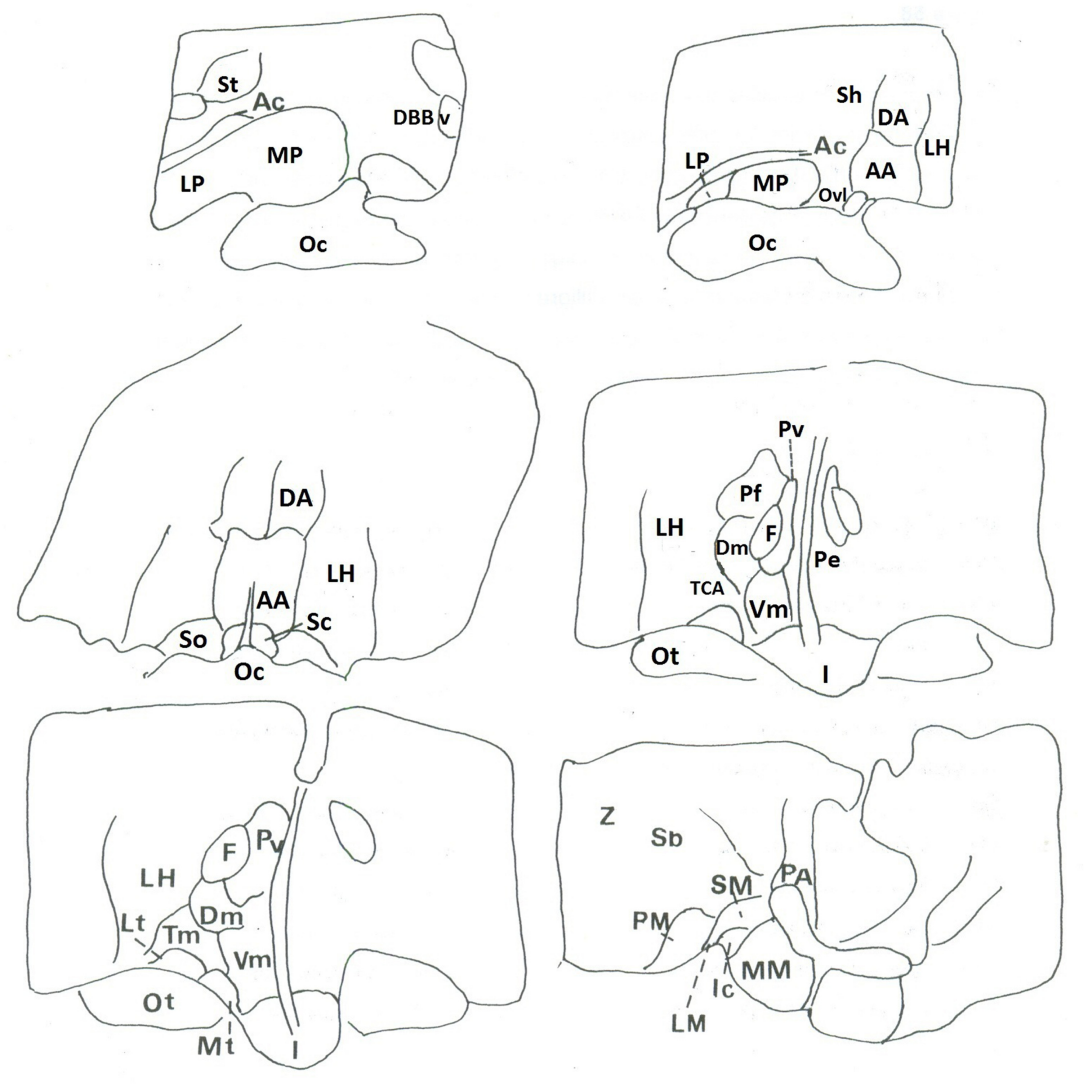
**Corresponding drawings and legends of structures of autoradiograms**.

**Figure 3:**
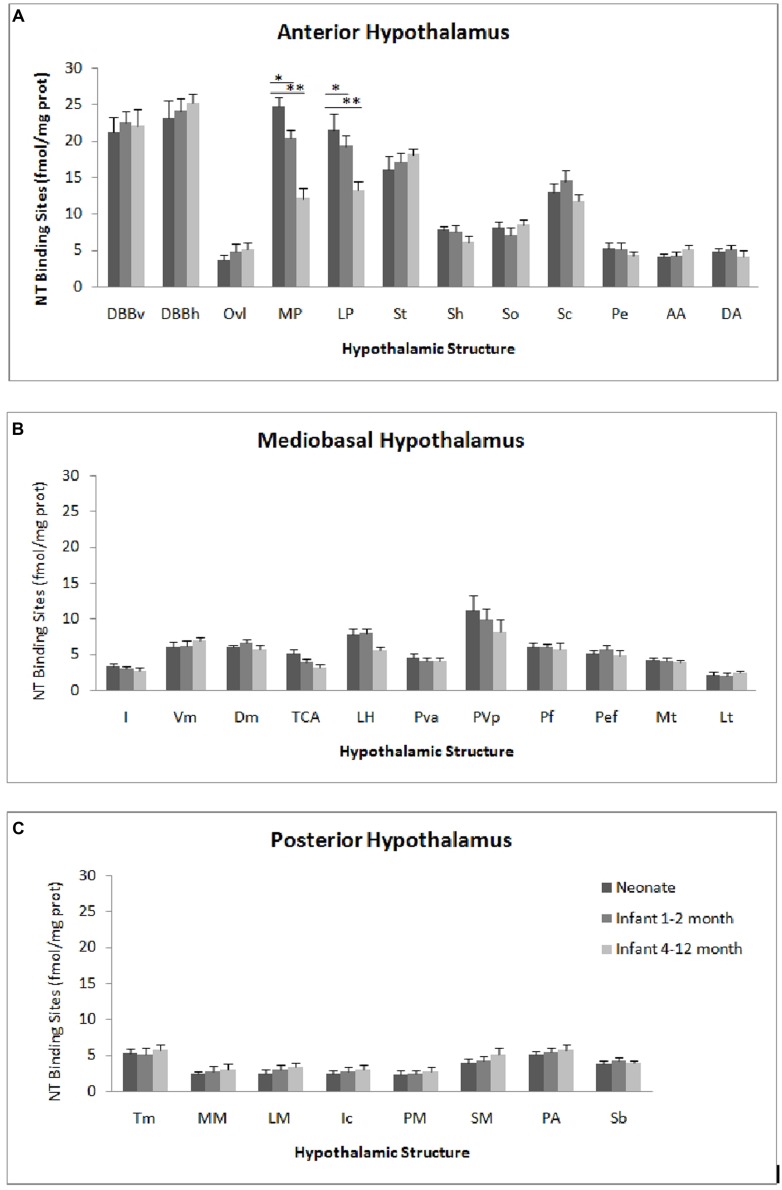
**Age comparison of NT receptor densities in the human hypothalamus during the peri- and the postnatal periods in the three hypothalamic levels: anterior (A), mediobasal **(B)** and posterior **(C)**.** The densities of binding sites are expressed as mean ± SEM of femtomoles of specifically bound per mg of protein (fmole/mg protein). *^,^**p < 0.001 neonate vs. infant (1–2 months) and neonate vs. infant 4–12 months respectively for medial preoptic area (MP). *^,^**p < 0.001 neonate vs. infant (4–12 months) and infant (1–2 months) vs. infant 4–12 months respectively for lateral preoptic area (LP).

The autoradiographic labeling was present throughout the rostrocaudal extent of the hypothalamic region in both neonates and infants. In all hypothalamic nuclei and areas analyzed, the non-specific binding, as determined in the presence of 1 μM NT_1-13_ was small throughout the concentration range (<10% of total binding). The distribution of NT binding sites throughout the human neonate/infant hypothalamus is shown in **Figure 1** and the corresponding drawings are reported in **Figure 2**. The detailed mapping has been realized in the three anatomical hypothalamic levels, i.e., anterior, mediobasal, and posterior hypothalamus. Quantification of ^125^I-NT binding site densities in the different hypothalamic structures is presented in the three histograms representing the comparison of NT binding sites density during the first postnatal year in the three hypothalamic levels (**Figure 3**).

### NEONATE

#### Anterior hypothalamus

The highest densities observed in the anterior hypothalamic level were present in the diagonal band of Broca and the medial and lateral preoptic areas (**Figures 1A** and **3A**). Dorsally, low to moderate autoradiographic labeling was revealed in the septo-hypothalamic nucleus, whereas the nucleus of the stria terminalis displayed a dense binding (**Figures 1A,B** and **3A**). At this level, low binding sites density is present in the anterior, dorsal and lateral hypothalamic areas as well as the organum vasculosum of lamina terminalis (**Figures 1B,C** and **3A**) whereas the suprachiasmatic nucleus shows moderate binding site densities (**Figures 1C** and **3A**) and the autoradiographic labeling was present throughout the entire structure. At the median level, low densities of NT binding sites were detected in the periventricular and paraventricular nuclei (**Figures 1D** and **3A**). The supraoptic nucleus presented a relatively moderate autoradiographic binding, mainly in the ventrolateral part (**Figures 1C,D** and **3A**). Finally, the fornix column which emerged at this level as well as the anterior commissure, showed no binding (**Figures 1A–D**).

#### Mediobasal hypothalamus

Generally, the mediobasal hypothalamic level presented relatively low autoradiographic labeling throughout its whole extent, particularly in the ventral portion. The infundibular, the ventromedial and the dorsomedial nuclei displayed equivalent low pattern of labeling (**Figures 1D,E** and **3B**). In contrast to the anterior hypothalamic level, the mediobasal part of the paraventricular nucleus as well as in the periventricular nucleus showed moderate NT binding site densities as (**Figures 1E** and **3B**). The parafornical nucleus also displayed moderate densities. In the posterior hypothalamic area, NT binding site density was moderate, existing principally in both its ventral and medial parts. The tuberal nuclei displayed low to moderate NT binding site density (**Figures 1D,E** and **3B**), the autoradiographic labeling was homogenous in both the medial and lateral portions. At the mediobasal hypothalamic level, the labeling present in the lateral hypothalamic area tended to relatively increase but remained in the low density average. Finally, no significant labeling was detected in the median eminence.

#### Posterior hypothalamus

In the transition zone between the mediobasal and the posterior hypothalamus, only low but significant NT binding site densities were detected in the premammillary nucleus and the surrounding areas (**Figures 1E** and **3C**). At the posterior level, the mammillary complex presented a diffuse and heterogeneous distribution of NT binding (**Figures 1F** and **3C**) with a low labeling in all the anatomical components: the lateral mammillary nucleus, the intercalatus nucleus, and the supramammillary nucleus (**Figures 1F** and **3C**). Dorsally, the posterior hypothalamic area, the subthalamicus nucleus and the zona incerta displayed very low densities (**Figures 1F** and **3C**), while the mamillothalamic tract and the Vicq d’Azyr fibers tract surrounding the medial mammillary nucleus were devoid of binding.

### INFANT

#### Anterior hypothalamus

The pattern of ^125^I-NT binding was quite similar to that observed for the neonate with binding sites density being in equivalent range for all anatomical components except for preoptic region where the densities were relatively moderate compared to neonate (20.1 ± 1.8 to 18.7 ± 1.4 in infants aged 1–2 months, vs. 25.1 ± 2.4 to 22.7 ± 2.1 in neonates, *p* < 0.001; 12.8 ± 2.0 to 13.1 ± 1.1 in infants aged 4–12 months, vs. 25.1 ± 2.4 to 22.7 ± 2.1 in neonates, *p* < 0.001; **Figure 3A**).

#### Mediobasal hypothalamus

All the structures analyzed in infant presented similar anatomical distribution of the autoradiographic labeling and equivalent densities of NT binding sites (**Figure 3B**).

#### Posterior hypothalamus

Similar topography of NT binding sites distribution was also present for this hypothalamic level in infant and neonate. In all the structures, the densities were slightly but not significantly, higher in infant than in neonate (**Figure 3C**).

No significant differences related to gender have been seen in all hypothalamic nuclei and areas in all the postnatal period investigated.

## DISCUSSION

To the best of our knowledge, there is no information concerning the developmental dynamics of NT binding sites in normal human hypothalamus. We investigated in the present study, the autoradiographic distribution and quantitation of NT receptors in hypothalamus of postmortem normal human hypothalamus during development. This completes our previous study, analyzing the overall distribution of NT binding sites in the adult human hypothalamus (Najimi et al., 2014). To date, three NT receptors with distinct functional and pharmacological properties have been identified: NT1, NT2, and nts3 (for review, see Le et al., 1996; Vincent et al., 1999). NT binds to all three receptors (Vincent et al., 1999). NT has lower affinity for the NT2 receptor compared with the NT1 receptor (Schotte et al., 1986; Mazella et al., 1996; Vita et al., 1998). In our study, biochemical characteristics of binding carried out on different hypothalamic levels, showed that ^125^I-NT bind to a high-affinity site with a *K*d value varying from 0.88 to 1.40 nM (*n* = 10). Interestingly, the affinity of the binding sites labeled for the radiolabeled ligand was very close to that reported previously for NT1 receptors in many species including human (Sarrieau et al., 1985; Szigethy et al., 1990; Benzing et al., 1992; Chigr et al., 1992; Zsürger et al., 1992). Furthermore, levocabastine, which has affinity for the NT2 receptor, did not inhibit specific binding of ^125^I-NT in competition experiments and autoradiographic studies. This insensitivity was observed for all hypothalamic nuclei and areas in all cases investigated. This is in favor of the presence of the high affinity subtype (NT1) in the human hypothalamus and particularly during the first postnatal year. Zsürger et al. (1992) reported the presence only of the high affinity type during the first postnatal year. The low affinity binding sites were absent during this period and they were first detected in15-month-old human brain. Thus, from their investigations made on hemispheric membrane preparations containing also the hypothalamic region, it appears clearly that the high affinity NT1 is the most abundant receptor subtype in fetuses and early postnatal stages, whereas later in development, the low affinity NT2 receptor is successively expressed during definite periods. Therefore, we could assume that under our experimental conditions and the postnatal period we investigated, that the binding sites of NT we observed; belong to the high affinity NT receptor subtype, commonly called NT1. In rodent brain, NT1 and NT2 exhibit markedly different patterns of expression during development. NT1 expression in most subcortical areas is detectable during the first postnatal week and increases progressively to reach adult levels by the third week (Palacios et al., 1988; Sato et al., 1992) whereas NT2 expression is detectable during the second postnatal week and increases to reach adult levels between the fourth and eighth weeks after birth (Schotte and Laduron, 1987; Sarret et al., 1998).

The NT binding sites we identified in the human hypothalamus were detected throughout the entire first postnatal period investigated and did not display different affinities for NT and analogs. Furthermore, individual kinetic affinities did not vary significantly, arguing for a stability of NT binding sites affinity during development. Interestingly, kinetics of NT binding, were equivalent to those we reported in adult hypothalamus (Najimi et al., 2014) suggesting that they are not age related. Such stability during postnatal development for NT binding sites has been reported previously in other human brain structures (Chigr et al., 1992; Zsürger et al., 1992).

Autoradiographic studies conducted in the developing hypothalamus have demonstrated that NT receptors are present in the entire rostrocaudal extent of the hypothalamic region and were exclusively present in gray matter. However, the labeling of NT binding sites across the hypothalamic region revealed somewhat several waves of receptors expression during development. Thus, the comparison of data obtained in preoptic region, at three age stages studied, from the neonate period to the 1 year postnatal age, showed gradual and significant decrease in NT binding sites density ( 24,2 ± 2.1 to 22.7 ± 2.1 in the neonate period vs. 12.8 ± 1.9 to 13.1 ± 1.1 in infant, *p* < 0.001). This progressive decrease, in density of NT binding sites, starts probably from the age of 23 weeks post-conceptional (presence of high densities in a fetus of 23 weeks of conceptional age, equivalent to the neonatal period, data not shown). This phenomenon continues throughout the first postnatal year and the densities decrease considerably to reach adult levels, but it is difficult with the sample we used to accurately determine the precise age when densities reach the level found in adults. Previous radioautographic studies have also highlighted the presence of more important densities of NT binding sites during the peri- and postnatal periods, in different brain structures in humans (Mailleux et al., 1990; Chigr et al., 1992; Zsürger et al., 1992). These differences in densities observed during the first postnatal period, could not be due to variation in the affinity. As discussed above, the affinity characteristics were remarkably stable during the first postnatal year. Also, the altered receptor binding, could not be due to differences in the postmortem delays (PMD). In accordance of this, previous studies in human brain with PMD’s up to 42 h (Palacios et al., 1991) or rat brain up to 48 h (Sadoul et al., 1984) showed no significant negative effect of PMD’s on NT binding. Furthermore, our previous studies in human did not relate any influence of this factor on NT receptor density (Sarrieau et al., 1985; Chigr et al., 1992; Najimi et al., 2014). Finally, the higher PMD of the sample investigated (34 h, case B) belong to the neonate group expressing high densities as well as for the lowest PMD (7 h, case J).

These differences in density observed with age (in the sense of decreasing density) could be the consequence of neuronal death involving these binding sites (Stanfield et al., 1982). This regressive process cell death occurs frequently during development in rat CNS is responsible for decreased levels of certain proteins in neurons after birth (Cowan et al., 1984). It has been postulated that these occurring changes may also result from elimination of neuronal connections during the fetal and the postnatal periods, leading to a differential stability of functional synaptic contacts. The elimination period modifying synaptic intercellular signals could lead to a repression of gene expression of NT receptor in hypothalamic neurons. The high expression of NT1 receptor early in development suggests that it may play a specific role in the establishment of neuronal circuitry (Boudin et al., 2000). The physiological significance of this transient expression of NT binding sites during neuronal maturation remains unknown. It may be suggested that NT acts as a regulatory peptide during ontogeny. Thus, the presence of NT receptor before the full establishment of neural networks suggests that it is involved in the regulation of developmental processes and maturation of these structures in the human hypothalamus. The high binding levels observed may be a consequence of the low levels of NT release and a compensatory up-regulation of receptors. In accordance of this, previous data report on the presence of relatively moderate amounts of NT immunoreactivity in human infant preoptic region, during the first postnatal month (Sakamoto et al., 1987).

As mentioned above and recall here, low binding sites density is observed during all the first postnatal year period in mammillary bodies compared to adults (Najimi et al., 2014). This is similar to that reported previously for somatostatin and benzodiazepine binding sites (Najimi et al., 1991, 2001). These results suggest an incomplete maturation of NT binding sites in these structures during the first postnatal period. We cannot exclude also that these hypothalamic structures have not yet completed their neuronal maturation (Kopp et al., 1992). Studies on older ages should reveal the precise period wherein the densities of binding sites begin to rise and reach levels observed in adults. It is also possible that these relatively low densities of NT binding sites are due to inhibitory control of NT on the endogenous expression of its correspondent receptor. Indeed, it has been shown in rat, that a blockade of neurotransmission for 5–9 days after the birth, with SR 48692 (NT antagonist), increases greatly the number of NT binding sites (Lépée-Lorgeoux et al., 1999). As previous studies reporting high and low amounts of endogenous NT in the human mammillary bodies of infant (Sakamoto et al., 1987) and adult (Langevin and Emson, 1982), respectively, one may speculate that endogenous NT levels would be responsible, at least in part, for the expression of NT binding, in the human hypothalamus, during peri- and postnatal phases. If in fact the reduced NT binding represents a physiologically relevant process, one plausible explanation would be that NT receptors in the hypothalamus during the postnatal period are down-regulated. Alternatively, the observed low levels of these binding sites could be explained by the fact that the synthesis of high-affinity NT receptors in fetuses and newborns is delayed in the posterior hypothalamus as has been reported for somatostatin receptors (Bologna and Leroux, 2000).

Taken together, it is tempting to postulate that the complex spatiotemporal pattern of NT binding sites expression in the developing hypothalamus in human, presumably results, at least partially, from the action of endogenous NT (Sakamoto et al., 1987) leading to an up or down regulation of NT receptors, as has been reported for rat brain (Palacios et al., 1988; Lépée-Lorgeoux et al., 2000). Nevertheless, and despite the well established modulatory role of the peptide on NT1 receptors in mature brain in rat (Azzi et al., 1996), it does not play a major role in establishing their developmental profile during the early postnatal period (Lépée-Lorgeoux et al., 2000). This could suggest that peptide release during this period, as well as connection formation, are probably not sufficient for the regulation of NT1 receptors by the endogenous ligand. It is therefore possible that other unknown mechanisms may explain these developmental changes. Thus, the progressive reduction in NT1 receptors density during the postnatal period in the human hypothalamus could coincide with normal maturational events characterized by programmed cell death of neurons bearing N receptors, or the influence of hypothalamic inputs on the expression of NT_1_ receptors. A reduction in NT binding may simply reflect a loss of synaptic connectivity (Stanfield et al., 1982; Cowan et al., 1984; Zecevic et al., 1989). On the contrary, in the posterior hypothalamus, the NT could be more involved in the maturational process of NT1 receptor. This could be corroborated by *in vitro* and *in vivo* experiments which have demonstrated a variety of effects of NT in the developmental processes such as the facilitation of dendritic outgrowth (Gandou et al., 2010), the regulation of growth factor secretion by astrocytes (Camby et al., 1996), the modulation of neuron sensitivity to glutamate (Ferraro et al., 2008) as well as important role during the onset of neuritic ingrowth (Staecker et al., 1996).

The characteristic of postnatal autoradiographic pattern of ^125^I-NT binding in the human hypothalamus is not unique to the neurotransmitter/neuromodulator receptor studied to date. Such a short-lasting expression of a particular receptor has been shown for vasoactive intestinal peptide (VIP) and benzodiazepine receptors in the hypothalamus during the first postnatal year (Najimi et al., 2001, 2006).

The actions of NT as a regulator of anterior pituitary secretions and food intake has been well characterized (Rostène and Alexander, 1997; Kalafatakis and Triantafyllou, 2011). Interestingly, the presence of considerable amounts of the peptide (Sakamoto et al., 1987) as well as NT binding sites (this study) is in favor of the direct control or modulation (by finely tuning feeding regulation; Brunetti et al., 2005) of these autonomic function in neonate/infant. This function is highly vital for the survival of the body during this sensitive period of life. Indeed, neonatal nutrition during critical periods plays a key role in the development of obesity and related metabolic pathologies such as cardiovascular disease and type II diabetes (McMillen and Robinson, 2005).

In summary, the results obtained in this study sustain the concept that the NTergic system participates in the development, differentiation, and maintenance of the human hypothalamus. As perspectives, the present work should be extended, by investigating, firstly the characteristics of NT binding sites during the other postnatal years (particularly the second postnatal year) to examine the eventual dynamics in binding site levels notably in the posterior hypothalamus. Secondly, as the sensitive levocabastine subtype is expressed at the 15th postnatal month in human brain, it would be very interesting to examine in these postnatal ages, if these binding sites subtype are present in the human hypothalamus.

## Conflict of Interest Statement

The reviewer, Dr. Valérie Compan declares that despite having collaborated with the authors, the review process was handled objectively. The authors declare that the research was conducted in the absence of any commercial or financial relationships that could be construed as a potential conflict of interest.
